# The effect of different administrations of testosterone therapy on adverse prostate events: A Bayesian network meta-analysis

**DOI:** 10.3389/fendo.2022.1009900

**Published:** 2022-11-07

**Authors:** Bin Zeng, Shi Qiu, Xingyu Xiong, Xingyang Su, Zilong Zhang, Qiang Wei, Lu Yang

**Affiliations:** ^1^ Department of Urology, Institute of Urology and National Clinical Research Center for Geriatrics, West China Hospital, Sichuan University, Chengdu, China; ^2^ Center of Biomedical Big Data, West China Hospital, Sichuan University, Chengdu, Sichuan, China

**Keywords:** hypogonadism, administrations, prostate safety, meta-analysis, systematic review, testosterone therapy

## Abstract

**Background:**

Hypogonadism has become a major cause endangering men’s health and quality of life all over the world. Testosterone Therapy (TT) is a widely accepted treatment for relieving hypogonadal symptoms. However, the effect of different administrations of TT on prostate safety is still unclear.

**Methods:**

We did a thorough search of PubMed, Embase and Cochrane Library to identify eligible studies up to January 2022. Randomized controlled trials (RCTs) and Cohort studies evaluating the impacts of using different formulations of TT on prostate parameters were included. Changes of prostate-specific antigen (PSA) level and prostate cancer (Pca) cases were used as the primary outcomes. Quality of individual studies was estimated by RoB_2_ (Cochrane tool for assessing the risk of bias in randomized trials) and the Newcastle-Ottawa scale (Tool for assessing non-RCTs). Certainty of evidence for each study was evaluated according to the evidence assessment criteria of the Oxford Evidence-based Medicine Center. Random-effect network meta-analysis(NMA)was performed based on the Bayesian model.

**Results:**

Thirty-five studies (30 RCTs and 5 Cohort studies) with 7,740 participants were included. TT administration led to fewer Pca patients (RR=0.62, 95%CI [0.39,0.99], I^2^=0%), while little decreasing in PSA level (MD=-0.05, 95%CI [-0.08, -0.02], I^2^=0%). The NMA revealed that compared with other formulations, the intramuscular injection was the most likely to rank first in decreasing Pca cases. The TT also resulted in more biopsy cases (RR=2.38, 95%CI [1.01,5.60], I^2^=0%). As for NMA, intramuscular injection also performed relatively better in fewer prostate biopsy cases compared with transdermal group.

**Conclusion:**

TT does not lead to abnormal PSA changes and increased risk of Pca in patients with hypogonadism or low testosterone level. Compared with other preparations of TT, intramuscular injection proved better in minimizing Pca cases and was more likely to result in fewer prostate biopsy cases.

## 1 Introduction

Male hypogonadism, a clinical syndrome caused by androgen deficiency, has become a major cause endangering men’s health and quality of life all over the world. It is roughly estimated that about a quarter of men are affected by androgen deficiency (1). Some studies have shown that testosterone levels in healthy people may also gradually decline with age (2, 3). Hypogonadism becomes more common with age grow and usually occurs in men over the age of 40, but not limited to this (4). Among middle-aged men, the incidence of hypogonadism ranges from 2.1% to 12.8% (5). It is reported that about half a million men are diagnosed with androgen deficiency every year in the United States, especially in people with certain diseases, such as obesity and diabetes (6). This common hypogonadism in middle-aged people is also known as late-onset hypogonadism (LOH). LOH usually induces many other diseases by reducing sex hormones, such as atherosclerosis, hyperglycemia, hypertension, and so on (7, 8).

Testosterone Therapy (TT) has become a widely accepted treatment to alleviate the symptoms of hypogonadism. However, there still remains many controversies about the safety of TT (9). One of the most controversial is the possible consequences for prostate safety risk (10). Traditional theories suggest that the presence of testosterone may affect or even promote the growth of prostate. Nevertheless, some studies have shown that testosterone treatment does not lead to histological changes in the prostate (11, 12). Marks et al (12) and Baillargeon et al (13) reported that TT does not increase the risk of Pca or lead to more aggressive prostate tumors. The EAU guidelines also pointed out that there is no evidence that testosterone therapy increases the risk of prostate cancer (4). Therefore, the association between TT and prostate safety risk remains unclear.

Previously, Pastuszak et al (14) conducted a study which compared the long-term effects of different testosterone preparations on men with hypogonadism. Inspired by their article, we try to explore the impact of TT on prostate safety risk from the perspective of different administrations of testosterone. Testosterone is currently available in gel, patch, intramuscular, oral and other preparations. They perform differently in the route of administration, pharmacokinetics and adverse events. Therefore, we can reasonably speculate that different administration methods of testosterone have different impacts on the prostate safety risk of TT. Nevertheless, there are few head-to-head RCTs to evaluate the prostate safety risk of different formulations, and network meta-analysis technology is needed to assess the comparison between preparations. In the absence of RCTs with direct pairwise comparison, network meta-analysis (NMA) is the most robust way to compare the interaction of different treatment groups in multiple compilation studies.

Therefore, the aim of this study is to make full use of the clinical data obtained from the included studies to conduct a NMA to assess the prostate safety risk of different administrations of TT and find out the hierarchical structure of them.

## 2 Methods

### 2.1 Search strategy

According to the Preferred Reporting Items for Systematic Reviews and Meta-analyses (PRISMA) reporting guideline and its extension for NMA, we conducted the systematic review and network meta-analysis (15, 16). Three main electronic databases (PubMed, Cochrane Library and Embase) were searched to find out potentially relevant research up to January 2022, with language restricted to English. We used the following MeSH terms for search: “Prostatic Neoplasms”, “Testosterone”, and “Hypogonadism”. The complete search strategy used for PubMed was shown in **Supplementary Table 1**. We also manually retrieved the reference lists of relevant studies and reviews. Two authors (BZ and XYX) independently reviewed the literature, and the inconsistencies were discussed and solved with the third author (SQ).

### 2.2 Selection criteria

**Figure 1:**
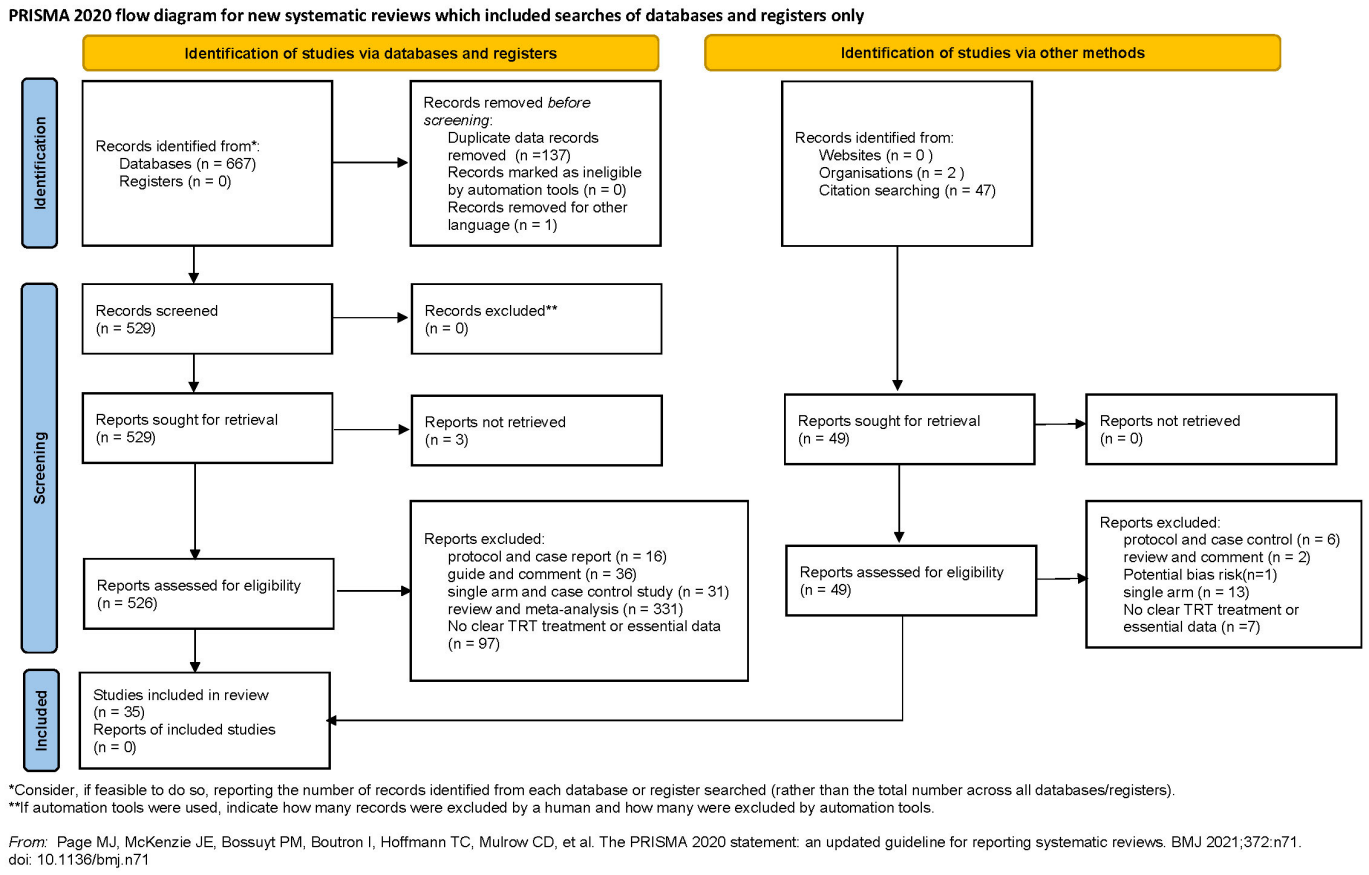
Preferred Reporting Items for Systematic Reviews and Meta-analyses flow diagram of study selection process.

The literature was reviewed by two separate authors independently (BZ and XYX). We evaluated the eligibility of studies using the population, intervention, comparator, outcome, and study (PICOS) method: (P) research involving population with hypogonadism or low testosterone level; (I) received different testosterone administrations (mentioned which administration was used); (C) group assigned with placebo or no TT was regarded as a comparator; (O) reporting one or more parameters of prostate safety; (S) RCTs or Cohort Studies. The selection process was shown in **Figure 1** based on the PRISMA flowchart.

### 2.3 Data extraction

The required clinical data were extracted by two separate reviewers from the included literature (BZ and XYX), including the following: author, year of publication, intervention measures, type of study, sample size, administration method, testosterone cut off level for study entry, exclusion criteria of prostate-specific antigen (PSA), median age, duration of study, dosage of the testosterone and the functional outcomes such as: Pca cases, PSA, prostate nodule, biopsy cases, abnormal PSA, International Prostate Symptom Score (IPSS) and prostate volume. Any inconsistencies were discussed and solved with the third reviewer (SQ).

### 2.4 Primary and secondary outcomes

As for the safety risk of prostate, we mainly evaluated it from two aspects: Pca and prostate growth. Referring to previous studies, we used the Pca cases, changes in PSA levels, prostate biopsy cases, prostate nodules and abnormal PSA to assess the risk of Pca (17). In terms of prostate growth, we used IPSS and prostate volume changes to evaluate. To ensure the reliability of the results, we used the two indicators which included most studies: the change of PSA level (27 studies) and Pca cases (19 studies) as the primary outcomes. And the rest indicators were used as the secondary outcomes in this study.

### 2.5 Study quality assessment

The risk of bias in each research was evaluated independently by two reviewers (BZ and XYX). According to the recommendations of the Cochrane manual, we selected RoB_2_, the most commonly used tool in randomized trials, as the tool to evaluate the risk of bias in RCTs (18). As for non-RCT studies, the Newcastle–Ottawa scale was used to assess the quality of included cohort studies (19). In this study, we believe that the total score ≧ 6 is high quality for cohort studies. Furthermore, consistent with previous studies, the certainty of evidence for each study was evaluated according to the evidence assessment criteria of the Oxford Evidence-based Medicine Center (20).

### 2.6 Consistency and sensitivity analysis

The NMA was based on the consistency assumption. We used the node-split method to verify the consistency between direct comparison and indirect comparison (21). The sensitivity analysis was conducted to examine the reliability of our findings. We excluded cohort studies which may be the source of heterogeneity in results and reanalyzed the clinical data without cohort studies. The results were compared with our previous findings to see whether the two results were consistent.

### 2.7 Data synthesis and statistical analyses

The continuous outcomes in our study were transformed into the same units of measurement and were pooled as mean difference (MD). And the relative risk (RR) was used to evaluate dichotomous variables. If the extracted data from the included studies were recorded in the median (interquartile range), we calculated the mean ± standard deviation (SD) according to the method described by Hozo et al. (22). We performed the pairwise meta-analysis using Review Manager v5.4 software to calculate the MD and RR of primary and secondary outcomes between different testosterone preparations and the control group. Given the possible potential heterogeneity, we used the Mantel-Haenszel (M-H) random-effects model for evaluation. The heterogeneity between included studies was estimated using chi-square (p < 0.05) and the I^2^ statistic (23). If I^2^ > 50%, we consider there may be moderate-to-high heterogeneity between studies (24).

The NMA was performed in a Bayesian hierarchical framework, and we used the random-effects model to evaluate the direct and indirect comparison between different testosterone formulations (25). In order to ensure the high level of evidence of NMA, we only used the data from RCTs for the NMA. And we used the Brooks-Gelman-Rubin method to evaluate the convergence (26). Intra-chain and inter-chain variances were compared by the potential scale reduction factor (PSRF), which was calculated in this method. If PSRF is close to 1, we consider that it has reached the approximate convergence (27). In the process of gradual convergence, there are four chains, the variance scaling factor was 2.5, simulation iterations were 100,000, tuning iterations were 20,000, and the thinning interval was 10. Rank probabilities were calculated for all different testosterone formulations and control group, and the surface under the cumulative ranking curve (SUCRA) is estimated by dividing the cumulative probability of all rankings by the number of rankings minus 1.

All statistical tests were 2-sided, and 95% confidence intervals (CIs) were reported. P < 0.05 was defined as statistically significant in this study. We used the “gemtc” “rjags” “meta” and “metafor” packages from R 4.1.2 (R project) (28) and Review Manager v5.4 software to conduct all the statistical analyses and forest plots.

## 3 Results

### 3.1 Study selection and network structure

We identified 716 articles for appraisal, including 49 studies which were searched from reference lists of relevant studies and reviews. After excluding the literature that does not meet the inclusion criteria, a total of 35 studies involving 7,740 participants were included. The detailed selection process was shown in **Figure 1**. In addition, the 35 included studies were consisted of 30 RCTs (11, 12, 29–56) and 5 cohort studies (14, 57–60).

**Figure 2:**
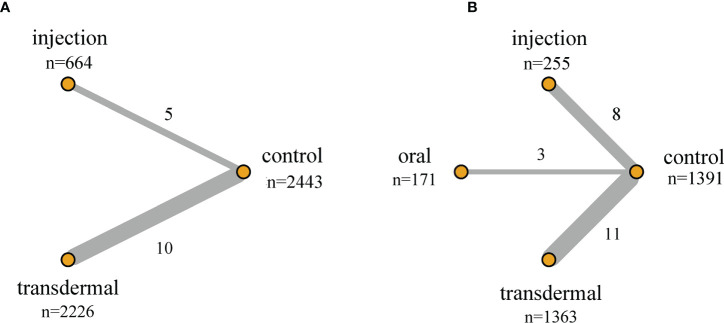
Network structure of the comparisons of primary outcomes for the Bayesian network meta-analysis. **(A)** Pca Cases; **(B)** PSA Level Changes. The size of the circles represents the sample size of each arm (in parentheses), and the thickness of the line represents the number of head-to-head studies (beside the line).

When evaluating Pca cases, abnormal PSA and prostate nodule for NMA, we found that only one literature with a value of 0 was included in certain preparation, which may lead to abnormal results. In order to assure the robustness of the results, we excluded these unstable studies for evaluation in our NMA. The network structure for Pca cases and PSA level changes were displayed in **Figure 2**. The size of the circle represents the sample size of each arm, and the thickness of the line represents the number of head-to-head studies. If there is no connection between circles, it indicates that there are no research with a direct comparison between the two preparations at present.

### 3.2 Characteristics of included studies

The characteristics of the whole included studies were shown in **Table 1**. According to different definitions, the baseline testosterone levels of all included 7,740 participants were in the range of testosterone deficiency or normal low levels. The average age of all the participants ranged from 44 to 80 years old. The duration of participants’ follow-up ranged from 1 month to 12 years after the first administration. In this study, there were three ways of TT administration to compare the primary and secondary results, including oral, intramuscular and transdermal. Of the 35 articles included, 16 studies (11, 30–32, 36–46, 58) compared transdermal testosterone with the control group (placebo or No testosterone), 13 studies (12, 33–35, 47–52, 55–57) compared intramuscular testosterone with the control group, 3 studies (29, 53, 54) compared oral testosterone with the control group, and 3 studies (14, 59, 60) compared intramuscular testosterone with transdermal testosterone. Among the participants, 171 participants were included in the oral testosterone group, 1,404 participants in the intramuscular testosterone group, and 2,750 participants in the transdermal testosterone group.

**Table 1:** Characteristics of included studies and participants.

				Sample size								
Study	Therapy in experimental group	Therapy in control group	Type of study	Experimental	Control	Administration method	Testosterone cut off level for study entry	Exclusion criteria of PSA	Age median/mean(y)	Duration(month)	Dosage	LE
Rhee (2021)	T	Placebo	RCT	19	23	O	——	PSA>4 ng/mL	69.5	6	40 mg/day	2
Wittert (2021)	T	Placebo	RCT	504	503	I	TT 14nmol/L	——	59.7	24	1000 mg per 6 weeks	1
Antonic (2020)	T	Placebo	RCT	28	27	I	TT 11 nmol/L or FT 220 pmol/L	PSA>4μg/L	60.2	24	1000 mg per 6 weeks	1
Cunningham (2019)	T	placebo	RCT	395	395	T*	TT 275 ng/dL	——	72.2	12	50mg/day	1
Brock (2016)	T	Placebo	RCT	354	356	T*	TT 300ng/dL	PSA>4ng/mL	55.3	4	60mg/day	1
Snyder (2016)	T	Placebo	RCT	395	395	T*	TT 275 ng/Dl	——	72.2	12	50mg/day	1
Thirumalai (2016)	T	Placebo	RCT	43	8	T*	TT normal	PSA>2 ng/mL.	44	3	12.5mg, 25mg, 50mg, 100mg, or 150mg/day	2
Hackett (2014)	T	Placebo	RCT	92	98	I	TT 12 nmol/L or FT 0.25 nmol/L	PSA>4mg/L	61.6	7.5	1000 mg per 6 weeks	1
Borst (2014)	T	Placebo	RCT	14	16	I	TT 300 ng/dL or BT 70 ng/dL	PSA>2.6 ng/mL	70	12	125mg per week	2
Del (2013)	T	placebo	RCT	13	16	I	BT 70 ng/dL	PSA>4 ng/mL	60.3	1	150 or 200 mg per 2 weeks	1
Hildreth (2013)	T	placebo	RCT	96	47	T*	TT 200–350 ng/dL	PSA above the age-adjusted normal level	66.5	12	50mg/day	2
Behre (2012)	T	Placebo	RCT	183	179	T*	TT 15 nmol/L and BT 6.68 nmol/L	PSA>4 ng/mL	62	12	50–75 mg/day	1
Jones (2011)	T	placebo	RCT	108	112	T*	TT 11nmol/L or FT 225 pmol/L	age-adjusted elevated PSA level	59.9	12	60mg/day	1
Kaufman (2011)	T	placebo	RCT	234	40	T*	TT 300 ng/dL	PSA>2.5 ng/mL	53.9	6.5	40.5mg/day	2
Aversa (2010)	T	Placebo	RCT	40	10	I	TT 11nmol/L or FT 250 pmol/L	age-adjusted elevated PSA level	57.8	24	1000mg per 12 weeks	2
Basaria (2010)	T	placebo	RCT	106	103	T*	TT 3.5—12.1nmol/L or FT 173 pmol/L	PSA>4 ng/mL	74	6	100mg/day	1
Kenny (2010)	T	placebo	RCT	69	62	T*	TT 350 ng/dL	PSA>6.5 ng/dL	77.1	24	5mg/day	2
Kalinchenko (2010)	T	Placebo	RCT	113	71	I	TT 12nmol/L or FT 225pmol/L	PSA>4μg/L	52.1	7.5	1000 mg per 6 weeks	2
Srinivas-Shankar (2010)	T	Placebo	RCT	130	132	T*	TT 12 nmol/L or FT 250 pmol/L	PSA>4ng/mL	73.8	6	50mg/day	2
Shigehara (2011)	T	Placebo	RCT	23	23	I	FT 11.8 pg/ml	PSA>2ng/ml	70.5	12	250 mg per 4 weeks	2
Emmelot-Vonk (2008)	T	Placebo	RCT	113	110	O	TT 13.7nmol/L	PSA above the age-adjusted normal level	67.2	6	80mg×2/day	2
Marks (2006)	T	Placebo	RCT	21	19	I	TT 300 ng/dL	PSA>10ng/mL	65.1	6	150mg per 2 weeks	2
Nair (2006)	T	Placebo	RCT	27	31	T*	BT 3.6nmol/L	PSA above the age-adjusted normal level	67.2	24	5mg/day	2
Amory (2004)	T	placebo	RCT	24	24	I	TT 12.1nmol/L	PSA>4 ng/mL	71	36	200mg per 2 weeks	2
Kenny (2004)	T	placebo	RCT	6	5	I	BT 128 ng/dL	PSA>4 ng/mL	80	3	200mg per 3 weeks	2
Steidle (2003)	T	placebo	RCT	307	99	T*	TT 10.4 nmol/L	PSA above the age-adjusted normal level	58	3	50 mg/day or 100 mg/day	2
Wittert (2003)	T	placebo	RCT	39	37	O	TT 8 nmol/L	PSA>5 ng/mL	68.5	12	80mg×2/day	2
Ferrando (2002)	T	placebo	RCT	7	5	I	TT 17 nmol/L	PSA>4μg/L	67.6	6	100mg/day	2
Kenny (2001)	T	placebo	RCT	24	20	T*	BT 4.44 nmol/L	PSA above the age-adjusted normal level	76	12	5mg/day	2
Simon D (2001)	T	placebo	RCT	6	6	T*	TT 3.4 ng/ml	PSA above the age-adjusted normal level	52.6	3	125mg/day	2
Saad (2020)	T	No T	cohort study	412	393	I	TT 12.1 nmol/L	——	60.6	144	1000mg per 12 weeks	2
Pastuszak (2015)	T(transdermal)	T(injection)	cohort study	121	57	I and T*	TT 350 ng/dL	——	50.3	36	50–100 mg/day	3
Pastuszak (2013)	T	No T	cohort study	103	50	T*	——	——	61	over 36	——	3
Rhoden (2006)	T(transdermal)	T(injection)	cohort study	33	25	I and T*	TT 300 ng/mL or FT 1.5 ng/dL	PSA>4 ng/mL	58.3	12	——	2
Guay (2000)	T(transdermal)	T(injection)	cohort study	16	25	I and T*	——	——	——	2 or 3	200mg per 2 weeks and 5mg/day	3

T, testosterone; T*, transdermal; I, injection; O, oral; TT, total testosterone; FT, free testosterone; BT, bioactive testosterone; PSA, prostate specific antigen; LE, level of evidence.

### 3.3 Assessment of study quality

The bias risk assessment results of the studies were provided in **Table 2** and **Table 3**. According to the recommendations of the Cochrane manual, we used the RoB_2_ tool to assess the risk of bias of all included RCTs. Among the 30 RCTs, six trials (11, 33, 35, 41, 49, 51) had some concerns about the bias in measurement of outcomes, most of which were caused by inappropriate measurement methods. In addition, there is a risk of missing outcome data bias in one literature (42), mainly because of the failure to assess the impact of missing data on the results. As for cohort studies, the Newcastle–Ottawa scale was used to evaluate the quality of included research. Of the five cohort studies, most were considered to be of high quality, and three studies (58–60) were regarded as low quality due to poor control of confounding factors and too short follow-up time. According to the evidence evaluation criteria of the Oxford evidence-based medicine center, we evaluated the evidence level of each included study, and the results were shown in **Table 1**.

**Table 2:** Quality assessment of individual RCT.

Author (publish year)	R	D	Mi	Me	S	O
Rhee (2021)	Low	Low	Low	Low	Low	Low
Wittert (2021)	Low	Low	Low	Low	Low	Low
Antonic (2020)	Low	Low	Low	Low	Low	Low
Cunningham (2019)	Low	Low	Low	Low	Low	Low
Brock (2016)	Low	Low	Low	Low	Low	Low
Snyder (2016)	Low	Low	Low	Low	Low	Low
Thirumalai (2016)	Low	Low	Low	Some concerns	Low	Some concerns
Hackett (2014)	Low	Low	Low	Some concerns	Low	Some concerns
Borst (2013)	Low	Low	Low	Low	Low	Low
Del (2013)	Low	Low	Low	Some concerns	Low	Some concerns
Hildreth (2013)	Low	Low	Low	Low	Low	Low
Behre (2012)	Low	Low	Low	Low	Low	Low
Jones (2011)	Low	Low	Low	Low	Low	Low
Kaufman (2011)	Low	Low	Low	Low	Low	Low
Aversa (2010)	Low	Low	Low	Low	Low	Low
Basaria (2010)	Low	Low	Low	Low	Low	Low
Kenny (2010)	Low	Low	Some concerns	Low	Low	Some concerns
Kalinchenko (2010)	Low	Low	Low	Low	Low	Low
Srinivas-Shankar (2010)	Low	Low	Low	Low	Low	Low
Shigehara (2010)	Low	Low	Low	Low	Low	Low
Emmelot-Vonk (2008)	Low	Low	Low	Low	Low	Low
Marks (2006)	Low	Low	Low	Low	Low	Low
Nair (2006)	Low	Low	Low	Low	Low	Low
Amory (2004)	Low	Low	Low	Low	Low	Low
Kenny (2004)	Low	Low	Low	Some concerns	Low	Some concerns
Steidle (2003)	Low	Low	Low	Low	Low	Low
Wittert (2003)	Low	Low	Low	Low	Low	Low
Ferrando (2002)	Low	Low	Low	Some concerns	Low	Some concerns
Kenny (2001)	Low	Low	Low	Some concerns	Low	Some concerns
Simon D (2001)	Low	Low	Low	Low	Low	Low

Risk of bias legend R, Bias arising from the randomization process; D, Bias due to deviations from intended interventions; Mi, Bias due to missing outcome data; Me, Bias in measurement of the outcome; S, Bias in selection of the reported result; O, Overall risk of bias.

**Table 3:** Quality assessment of individual cohort study.

Author (publish year)	Selection				Comparability	Outcome			total
	exposed cohort	non exposed cohort	Ascertainment of exposure	outcome	Comparability	Assessment of outcome	follow-up	Adequacy of follow up of cohorts	
Saad (2020)	1	1	1	0	2	1	1	1	8
Pastuszak (2015)	1	1	1	0	0	1	1	1	6
Pastuszak (2013)	1	0	1	0	0	1	0	0	3
Rhoden (2006)	1	1	0	1	0	1	0	1	4
Guay (2000)	1	1	0	0	0	1	0	0	3

### 3.4 Results of primary outcomes

#### 3.4.1 Pca cases

**Figure 3:**
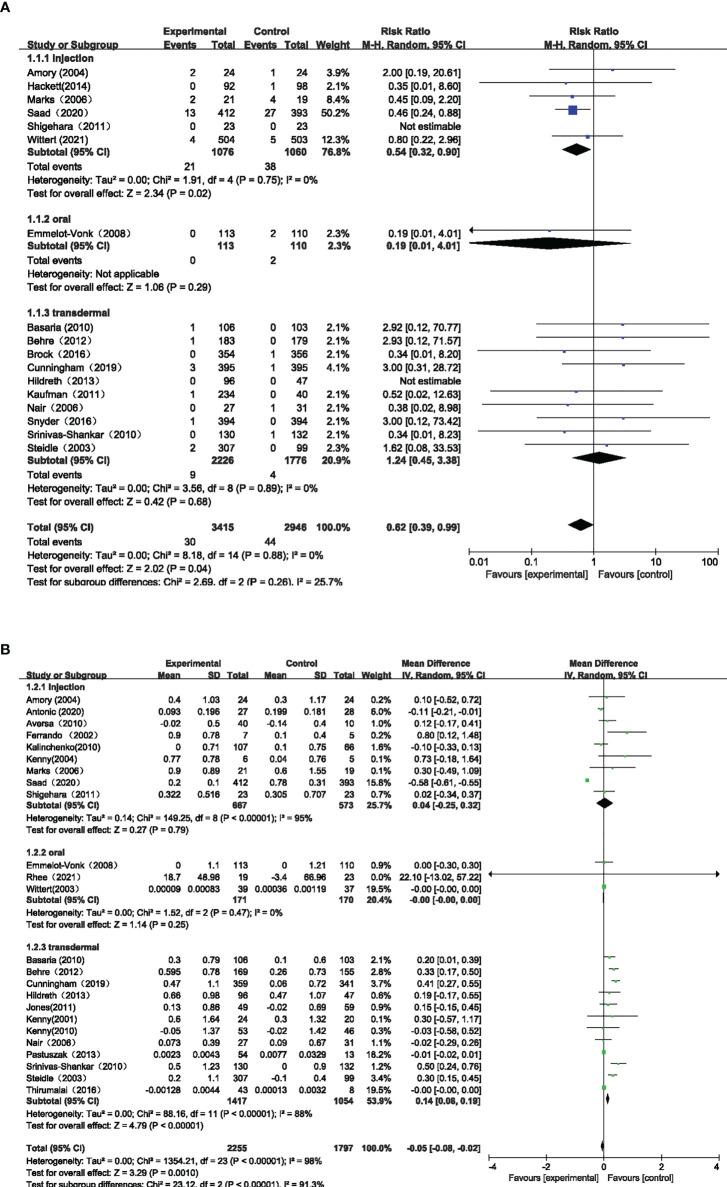
Pairwise Meta-analysis for primary outcomes. **(A)** Pca Cases; **(B)** PSA Level Changes.

Seventeen studies with 6,361 participants and three testosterone preparations, constituted the pairwise meta-analysis to evaluate the Pca cases. The pooled results suggested that the use of TT could lead to fewer Pca cases than the control group (RR=0.62, 95%CI [0.39,0.99], I^2^ = 0%). Comparing the effects of different testosterone preparations and the control group on the risk of Pca, we found that except for intramuscular testosterone group showed fewer cases of Pca (RR=0.54, 95%CI [0.32,0.90], I^2 =^ 0%), the oral group (RR=0.19, 95%CI [0.01,4.01]) and transdermal group (RR=1.24, 95%CI [0.45,3.38], I^2 =^ 0%) where there was no significant difference compared with the control group in Pca cases(**Figure 3A**).

There were 15 RCTs with 5,333 participants and two testosterone preparations included in the NMA. Since in the oral testosterone group there was only one study with a value of 0, which would lead to abnormal results in the evaluation. For the sake of robust results, we excluded the oral group for evaluation. The intramuscular injection group accounted for 12.5% of the data, the transdermal group accounted for 41.7%, and the remaining 45.8% were in the control group (**Figure 2A**). The NMA results were displayed in **Table 4A**. We used the staircase tables to evaluate the effect of intramuscular injection, transdermal and control groups on the Pca cases. Although there was no significant difference among intramuscular injection, transdermal and control groups, intramuscular injection could show the trend of leading fewer Pca cases. According to Bayesian model, we used SUCRA to rank the probability that each testosterone preparation ameliorated the risk of Pca. The results showed that the probability of intramuscular injection, transdermal and control groups were 18%, 84% and 48%, respectively. And owing to the reverse scaled outcome of Pca cases (where lower values indicate a better outcome), the intramuscular injection was the most likely to rank first in decreasing Pca cases (**Supplementary Figure 1A**).

**Table 4:** Network Meta-analysis for RR of Prostate Cancer and MD of PSA Level Change.

A				B			
**control**	0.65 (0.19, 2.08)	1.74 (0.49, 7.06)		**control**	0.11 (-0.05, 0.30)	0.00 (-0.26, 0.26)	0.22 (0.10, 0.34)*
1.54 (0.48, 5.23)	**injection**	2.68 (0.47, 17.50)		-0.11 (-0.30, 0.05)	**injection**	-0.11 (-0.45, 0.19)	0.10 (-0.12, 0.31)
0.57 (0.14, 2.03)	0.37 (0.06, 2.12)	**transdermal**		-0.00 (-0.26, 0.26)	0.11 (-0.19, 0.45)	**oral**	0.22 (-0.07, 0.51)
				-0.22 (-0.34, -0.10)*	-0.10 (-0.31, 0.12)	-0.22 (-0.51, 0.07)	**transdermal**
RR of Prostate Cancer				MD of PSA Level Change			

*Indicates the presence of statistical significance.

The color shading in this table is used to highlight the results of indirect comparison of different testosterone preparations, which is consistent with the relevant literature of the Lancet (https://doi.org/10.1016/S0140-6736(21)01640-8).

#### 3.4.2 PSA level changes

Twenty-four studies involving 4052 participants and three testosterone preparations, constituted the pairwise meta-analysis to assess changes in PSA levels. The pooled results suggested that the use of TT could result in little decrease in PSA level compared with the control group (MD=-0.05, 95%CI [-0.08, -0.02], I^2^ = 98%). Comparing the effects of different testosterone preparations and the control group on the changes of PSA level, we found that the transdermal group showed minute PSA increase ((MD=0.14, 95%CI [0.08,0.19], I^2^ = 88%), while the injection group (MD=0.04, 95%CI [-0.25,0.32], I^2^ = 95%) and the oral group (MD=-0.00, 95%CI [-0.00,0.00], I^2^ = 0%) showed that there was no significant statistical difference compared with the control group. (**Figure 3B**)

In the NMA, there were 22 RCTS with 3180 participants and three testosterone preparations included. The transdermal group accounted for 42.9% of the data, the injection group accounted for 8%, oral group accounted for 5.4% and the remaining 43.7% were in the control group (**Figure 2B**). The results of NMA were shown in the staircase **Table 4B**. In the absence of evidence of direct comparison between the oral, injection and transdermal groups, we used the NMA to produce an indirect comparison between them. The results indicated that the transdermal group showed more PSA changes than the control group (MD=0.22, 95%CI [0.10,0.34]) and there was no significant difference among the remaining groups. In the same way, we calculated the ranking probability of each group according to the Bayesian model. Similarity, due to the reverse scaled outcome of PSA changes, we found that the oral group had the highest probability of ranking first in ameliorating PSA level changes (**Supplementary Figure 1B**). The SUCRA indicated that the probability of oral, intramuscular injection, transdermal and control groups were 25%, 69%, 88% and 18%, respectively.

### 3.5 Results of secondary outcomes

A total of 29 studies evaluated secondary outcome indicators. **Supplementary Figure 2** displayed the results of our pairwise meta-analysis for the efficacy of different administrations of testosterone compared with the control group.

In terms of Pca risk, 16 studies evaluated abnormal PSA, 10 studies assessed prostate biopsy, and 6 studies evaluated prostate nodules. Our results indicated that the prostate biopsy was the only one where there was statistical difference between TT and the control group (RR=2.38, 95%CI [1.01,5.60], I^2 =^ 0%). The comparison of effects of different testosterone preparations and control group on prostate biopsy indicated that no significant difference was found in both transdermal group (RR=2.44, 95%CI [0.97,6.13], I^2^ = 0%) and intramuscular injection group (RR=2.00, 95%CI [0.19,20.61]) compared with the control group. Subsequently, in the absence of evidence of direct comparison between the injection and transdermal groups, we conducted the NMA of prostate biopsy to generate an indirect comparison between them. The results suggested that except for the comparison between the transdermal group and the control group (control vs transdermal, RR=0.16, 95%CI [0.03,0.62]), there was no significant difference between other groups (**Supplementary Table 2**). The control group had the highest probability of ranking first in fewer biopsy cases, and injection was more likely to perform better compared with transdermal group.

As for the risk of prostate growth, 12 studies evaluating IPSS and 10 studies evaluating prostate volume were included. In the pairwise meta-analysis, the pooled results suggested that the use of TT had no significant effect on IPSS (MD=-0.37, 95%CI [-3.45,2.71], I^2^ = 99%) and prostate volume (MD=3.35, 95%CI [-0.25,6.95], I^2^ = 95%) compared with control group.

### 3.6 Sensitivity analysis

We excluded cohort studies which may be the source of heterogeneity to perform the sensitivity analysis. After excluding all cohort studies, there were 30 RCTs remained. Since the studies evaluating prostate biopsy and prostate nodules are all RCTs, the results of sensitivity analysis were consistent with previous results. In pairwise meta-analysis, sensitivity analysis showed that the effect of TT on PSA level (MD=0.01, 95%CI [-0.00,0.02], I^2^ = 80%) was not statistically different from that of the control group, and the use of TT had no significant effect on Pca cases compared with the control group (RR=0.85, 95%CI [0.44,1.62], I^2^ = 0%), which was different from previous results. Comparing the effects of different testosterone preparations and control group on the changes of PSA level, we found that there was no statistical difference between oral and control group (MD=-0.00, 95%CI [-0.00,0.00], I^2^ = 0%), intramuscular injection and control group (MD=0.05, 95%CI [-0.12,0.21], I^2^ = 45%), except that the transdermal group showed little PSA increase (MD=0.22, 95%CI [0.07,0.37], I^2^ = 89%) (**Supplementary Figure 3**). As for the other outcome indicators, the results of sensitivity analysis were consistent with our previous results. In general, according to the results of sensitivity analysis, our analysis results are relatively robust.

## 4 Discussion

Up to now, the controversy about the safety of prostate treated with testosterone has not been resolved. EAU Guidelines on Sexual and Reproductive Health (2022) (4)states that testosterone treatment has historically raised concerns about the possibility of benign prostatic hyperplasia (BPH) associated with prostate growth, but a large number of studies have no evidence to prove any significant association between TT and abnormal prostate growth and Pca events. In addition, it is unclear how different testosterone administrations affect prostate safety, and which testosterone preparations are better. Therefore, we conducted an indirect and direct comparison of the effects of different testosterone preparations on prostate safety in a network meta-analysis. As far as we know, this study is the first NMA to assess the impact of different testosterone preparations on prostate safety risk.

Our findings suggested that the use of TT was associated with fewer Pca cases, little decreased PSA level and more prostate biopsy cases, while there was no significant difference between TT and the control group in other outcomes. However, considering that the unit of PSA is ng/ml, we believe that this small difference caused by TT is of no clinical significance, so we can hold that TT will not significantly affect the change of PSA level. No matter compared with the control group or other testosterone preparations, injection testosterone all had the greater potential to perform better in decreasing Pca cases. Although compared with other groups, the intramuscular injection group had the longest follow-up time (mean duration: 22.4 months) which indicates more likely to observe more Pca cases, this also indirectly shows the robustness of our results from another point of view. We believe that the difference between sensitivity analysis and our previous Pca results is also due to the same reason. The average follow-up time of included RCTs (mean duration:11.3 months) is even less than one year, which may lead to an unreliable conclusion that there is no significant relationship between TT and the incidence of Pca. As for the prostate biopsy, our results indicated that the control group had the highest probability to rank first in ameliorating prostate biopsy cases. Although TT may be associated with more biopsy cases, injection testosterone was relatively better than transdermal group in terms of fewer prostate biopsy cases. For why intramuscular injection seems to be more effective in protecting prostate, we speculate that it may be caused by the following reasons: 1. The pharmacokinetics of the three preparations are different. Compared with oral and transdermal testosterone administrations, intramuscular injection can maintain a stable high circulating testosterone level for a long time, so as to better protect the prostate (61). 2. Previous evidence showed that obesity is associated with an increased risk of prostate cancer (62). Intramuscular injection is more beneficial to muscles generation, which can more effectively reduce the risk of obesity and thus lower the incidence of prostate disease, compared with oral and transdermal testosterone (63).

Our results regarding the impact of TT on prostate safety are consistent with previously published studies on this topic. As for the effect of TT on prostate growth, according to traditional theory, prostate growth depends on the presence of androgens, so it has been suggested that TT may increase prostate volume (64). However, some meta-analysis studies have shown that TT has no significant effect on prostate volume and lower urinary tract symptoms (LUTS). In a meta-analysis performed by Guo et al (65), which involved 16 trials and 1,921 participants, the reviewers found that there was no significant change in IPSS levels in the participants who received the TT compared with the placebo group (MD=0.01, 95%CI [-0.37,0.39], P=0.96). Another meta-analysis involving 51 trials yielded similar results, showing no statistical differences in IPSS and lower urinary tract symptoms between participants treated with TT and the control group (66). This may be explained by Morgentaler and Traish’s “saturation hypothesis” (67), that is when androgen receptors in the human prostate are “saturated” with circulating androgens, the prostate does not grow in response to external androgen supplementation. Several large clinical trials have also validated our results (68, 69). Regarding the Pca risk of TT, some previous meta-analyses have also been consistent with our results. Kang et al (70) conducted a meta-analysis including 15 clinical trials to find out the correlation between TT and the risk of Pca. Their results indicated that using testosterone did not increase PSA levels, and TT was not significantly associated with the elevated PSA, which was in accordance with our results. Similar results have been found in some large clinical trials, which revealed that the incidence of Pca in the population receiving TT was always lower than the incidence rate reported in the general population (71, 72). This phenomenon of using testosterone may protect the prostate could be explained by the hypothesis proposed by xu et al (73). They believed that the significant decrease of testosterone level may play an important role in adverse prostate events, and stable testosterone level can significantly reduce the incidence of prostate disease. In addition, as mentioned above, testosterone can promote the formation of fat free tissue and reduce obesity rate, so as to protect the prostate.

Although no previous studies have conducted a network meta-analysis of the effects of various testosterone administrations on prostate safety yet, some systematic reviews of the currently available evidence for direct comparison between them have been conducted. Cui et al (74) conducted a systematic review of the effects of TT on prostate growth in 2013, and compared performance differences between different administrations. Their results suggested that TT did not increase the risk of prostate growth in either the short or long term, regardless of the administration method, which was consistent with our results. Additionally, Cui et al. also explored the effect of TT on Pca risk in 2014 (17). Their results of meta-analysis suggested that TT was safe in the short term and did not contribute to the development of Pca, regardless of administrations. Although the authors suggested that the study’s failure to detect an increased risk of Pca in TT group may be due to the fact that the included trials monitored the participants closely and withdrew those participants when their Pca risk was suspected. Nevertheless, we believe that this is inevitable, and we included more studies (our study vs Cui et al:23 vs 8) evaluating the impact of TT on Pca events, leading to different results compared with them.

A total of 30 RCTs and 5 cohort studies were included in this network meta-analysis. According to the quality assessment scale we used, most studies were of high quality. From a clinical perspective, the results of this study provide a new idea for further exploring the relationship between TT and prostate safety risk. The results of sensitivity analysis are basically consistent with those of our study, indicating that our results are robust and reliable.

Nevertheless, there are some limitations in our research. Firstly, there was some heterogeneity in testosterone dose, baseline PSA levels, and so on among the participants included in the study. For example, the dosage and timing of testosterone administration varied from study to study, which may affect the final outcomes of our analysis. Although subgroup and sensitivity analyses were conducted to assess the quality of the results, heterogeneity cannot be completely avoided. Secondly, most of the participants in our analysis were the elderly, and only in one study (11) the average age of the population was 44 years old. More research on middle-aged people should be included to make the analysis more representative. Thirdly, we have three included studies (44, 49, 51) with a total number of participants less than 20 in our analysis. Too few participants may affect the robustness of the analysis results. Finally, although studies included in our current analysis were followed for a relatively longer period than some previous studies, since Pca often takes 7-10 years to shift from inert to aggressive (75), more long-term follow-up data are still needed to better evaluate the impact of TT on Pca risk.

## 5 Conclusions

TT is an effective treatment for patients with hypogonadism. As for the fear that testosterone treatment may result in abnormal prostate growth and Pca risk, however, our research shows that TT does not cause abnormal prostate growth, and may even reduce the risk of Pca. In terms of the preparation type of TT, intramuscular seems to be more prominent in reducing the risk of Pca, and TT will not increase the risk of abnormal prostate growth regardless of the administration method. Further high-quality clinical research are required to confirm this observation.

## Data availability statement

The raw data supporting the conclusions of this article will be made available by the authors, without undue reservation.

## Author contributions

LY and QW were responsible for the idea and design of the study. BZ, XX and SQ did the analysis and interpreted the results of the study. XS and ZZ searched the databases and extracted clinical data from the included studies. BZ and XX wrote the first draft of this manuscript. SQ, LY and QW revised the manuscript and wrote the final version. All authors contributed to the article and approved the submitted version.

## Funding

The work was supported by National Natural Science Foundation of China (Grant No.81902578, 81974098), China Post-docotral Science Foundation (2017M612971), Post-doctoral Science Research Foundation of Sichuan University (2020SCU12041), Post-Doctor Research Project, West China Hospital, Sichuan University (2018HYBH085), National Clinical Research Center for Geriatrics, West China Hospital, Sichuan University (Z2018C01).

## Conflict of interest

The authors declare that the research was conducted in the absence of any commercial or financial relationships that could be construed as a potential conflict of interest.

## Publisher’s note

All claims expressed in this article are solely those of the authors and do not necessarily represent those of their affiliated organizations, or those of the publisher, the editors and the reviewers. Any product that may be evaluated in this article, or claim that may be made by its manufacturer, is not guaranteed or endorsed by the publisher.
