# Supplementary material for: The effect of different administrations of testosterone therapy on adverse prostate events: A Bayesian network meta-analysis
Source: Front Endocrinol (Lausanne). 2022 Nov 7;13:1009900. doi: 10.3389/fendo.2022.1009900 (PMC9678341; doi:10.3389/fendo.2022.1009900)
Supplement: Supplementary file 1 [file DataSheet_1.docx]

**Supplementary Materials**

**Supplementary Table 1**. Search strategy used in this network meta-analysis

**Search Strategy for PUBMED, EMBASE, and The Cochrane Library**

**Hypogonadism**

1. Hypogonadism [MeSH Terms]
2. 'Hypogonadism, isolated hypogonadotropic'[Title/Abstract]
3. 'Hypogonadism, hypogonadotropic'[Title/Abstract]
4. 'Hypogonadism, hypergonadotropic'[Title/Abstract]
5. 'Hypogonadotropic hypogonadism'[Title/Abstract]
6. 'Hypergonadotropic hypogonadism'[Title/Abstract]
7. Hypogonadal [Title/Abstract]
8. Hypogonadotropic [Title/Abstract]
9. OR/1-8

**Prostate cancer**

1. Prostatic Neoplasms [MeSH Terms]
2. Prostate Neoplasm*[Title/Abstract]
3. Prostatic Cancer*[Title/Abstract]
4. Prostate Cancer*[Title/Abstract]
5. Prostate tumor*[Title/Abstract]
6. Prostatic tumor*[Title/Abstract]
7. OR/10-16

**Testosterone Therapy**

1. Testosterone [MeSH Terms]
2. Testosterone [Title/Abstract]
3. OR/17-18

**Conclusion**

#9 AND #16 AND #19

**Supplementary Table 2.** Network Meta-analysis for Secondary outcomes

| **control** | 2.19 (0.12, 85.04) | **6.20 (1.62, 33.92) *** |
| --- | --- | --- |
| 0.46 (0.01, 8.57) | **injection** | 2.79 (0.06, 95.08) |
| **0.16 (0.03, 0.62) *** | 0.36 (0.01, 16.50) | **transdermal** |

**B**

**A**

| **control** | 1.26 (0.32, 5.44) | 1.62 (0.47, 7.60) | 1.78 (0.89, 3.85) |
| --- | --- | --- | --- |
| 0.80 (0.18, 3.11) | **injection** | 1.29 (0.20, 10.74) | 1.40 (0.29, 7.02) |
| 0.62 (0.13, 2.11) | 0.78 (0.09, 4.90) | **oral** | 1.09 (0.20, 4.62) |
| 0.56 (0.26, 1.13) | 0.71 (0.14, 3.44) | 0.91 (0.22, 5.06) | **transdermal** |

| **control** | 5.76 (-0.42, 11.84) | 1.12 (-12.89, 15.33) | 0.16 (-10.50, 10.97) |
| --- | --- | --- | --- |
| -0.02 (-1.96, 2.01) | **injection** | -4.60 (-20.07,10.91) | -5.63 (-17.95, 6.61) |
| -0.21 (-3.94, 3.49) | -0.20 (-4.44, 3.98) | **oral** | -1.01 (-18.39, 16.88) |
| -0.34 (-2.28, 1.33) | -0.33 (-3.15, 2.19) | -0.13 (-4.47, 3.89) | **transdermal** |

**C**

**D**

| **control** | 0.95 (0.07, 13.50) | 0.96 (0.08, 11.39) |
| --- | --- | --- |
| 1.05 (0.07, 15.31) | **oral** | 1.06 (0.03, 34.64) |
| 1.04 (0.09, 12.46) | 0.94 (0.03, 37.51) | **transdermal** |

**A:** RR of abnormal PSA cases; **B:** RR of Prostate biopsy; **C:** MD of IPSS (below diagonal) and Prostate volume (above diagonal)；**D**: RR of Prostate Nodule. * Indicates the presence of statistical significance.

**
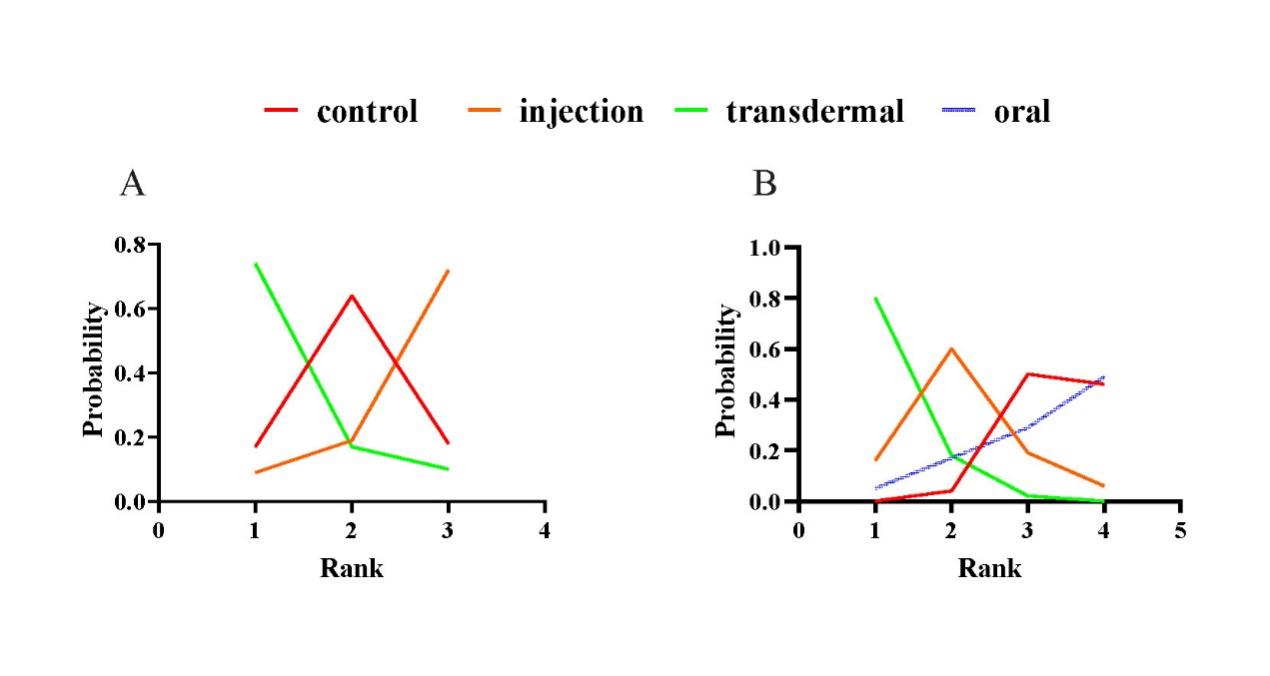
Supplementary Figure 1.** Ranking probability of included groups for primary outcomes.

1. Pca Cases; (B) PSA Level Changes

Owing to the reverse scaled primary outcomes (where lower values indicate a better outcome), the lowest ranking in the figure suggests the best outcome.

Owing to the reverse scaled primary outcomes (where lower values indicate a better outcome), the lowest ranking in the figure suggests the best outcome.

**
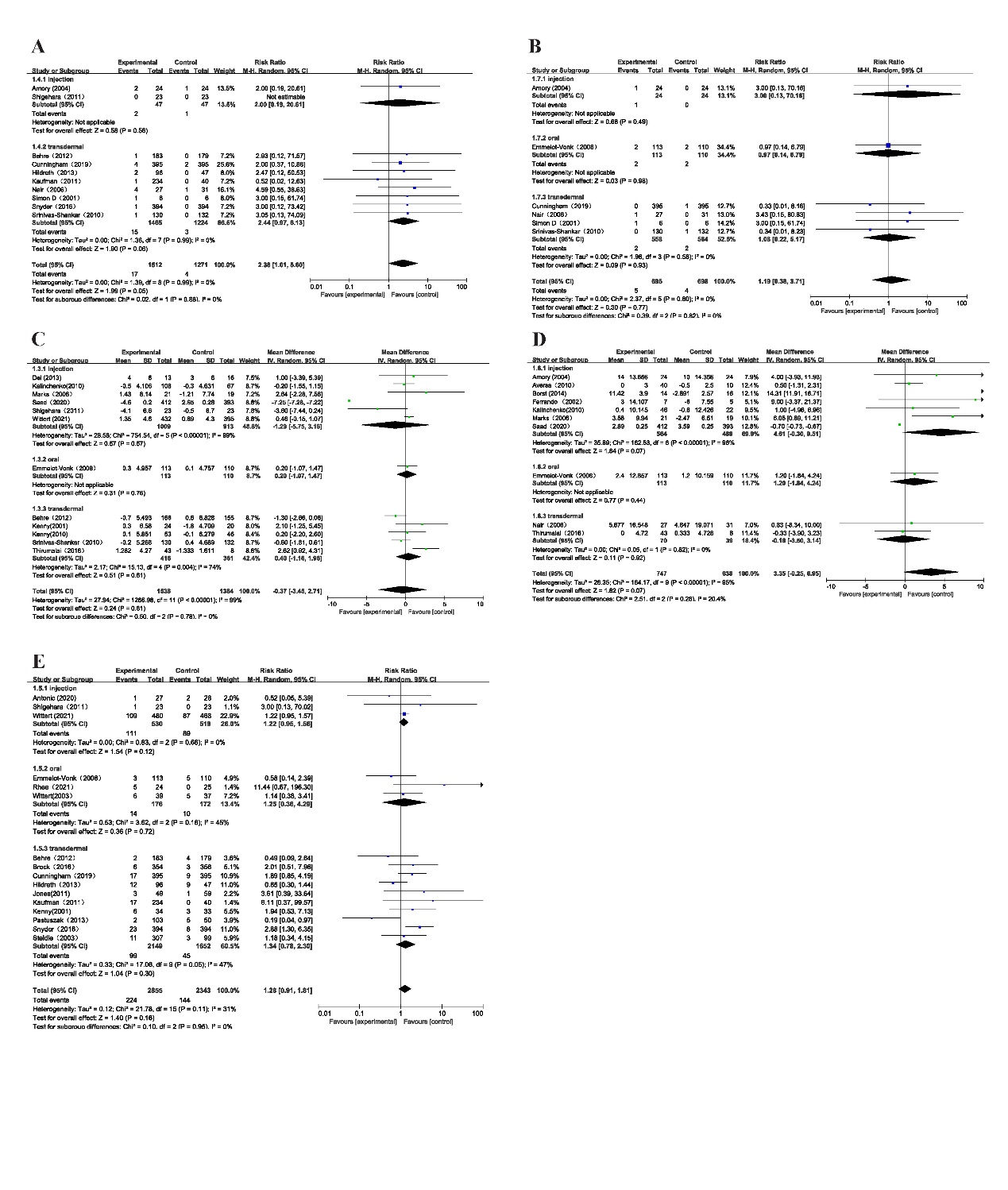
Supplementary Figure 2.** Pairwise Meta-analysis for secondary outcomes.

(A) Prostate Biopsy Cases; (B) Prostate Nodule Cases; (C) IPSS (international prostate symptom score); (D) Prostate Volume Changes; (E) Abnormal PSA Level Cases

**Supplementary Figure 3.** Pairwise Meta-analysis for primary outcomes of sensitivity analysis.

**
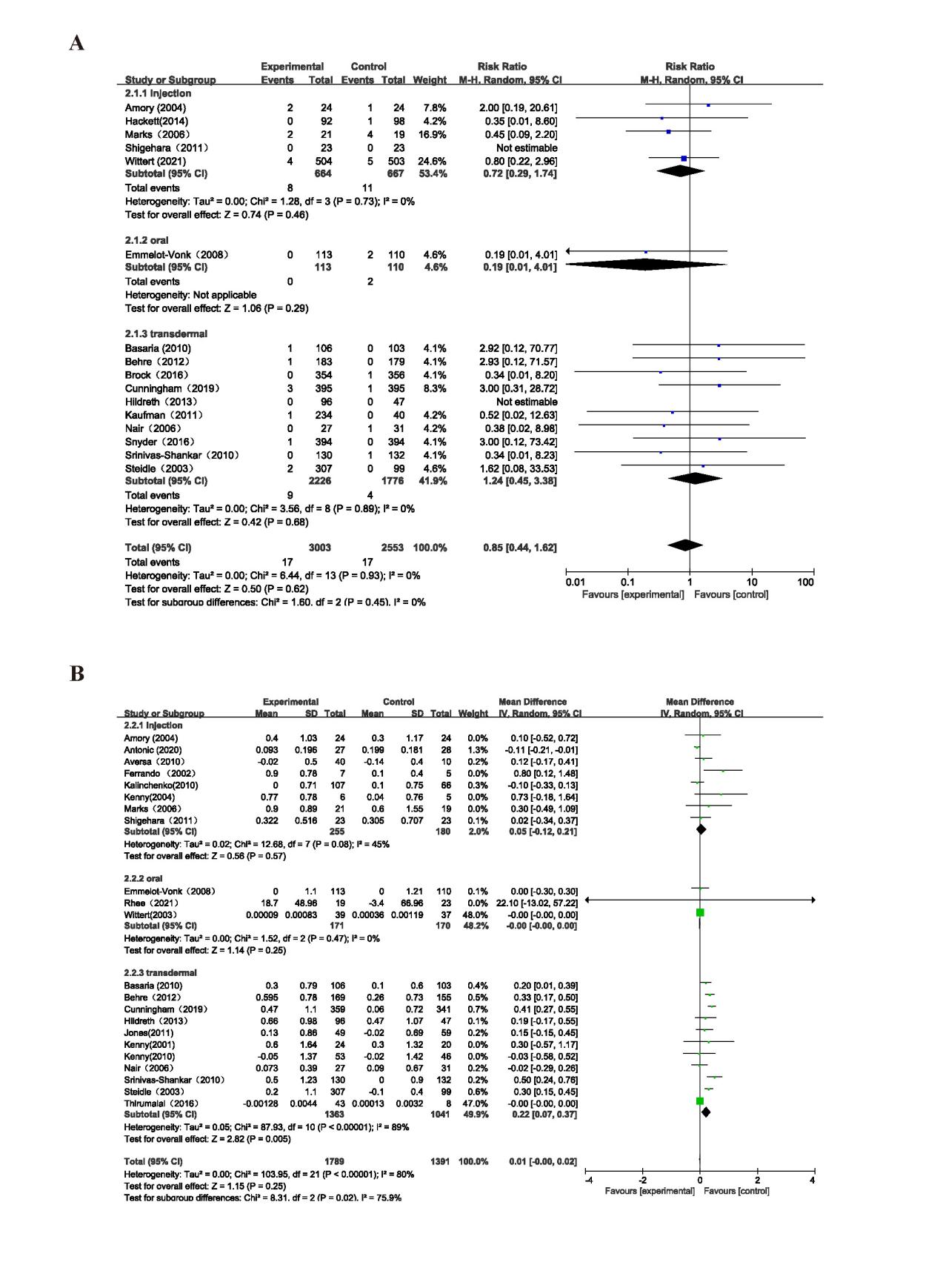
**

(A) Pca Cases; (B) PSA Level Changes
